# The sound of surgery-development of an acoustic trocar system enabling laparoscopic sound analysis

**DOI:** 10.1007/s11548-024-03183-2

**Published:** 2024-06-03

**Authors:** Daniel Ostler-Mildner, Luca Wegener, Jonas Fuchtmann, Hubertus Feussner, Dirk Wilhelm, Nassir Navab

**Affiliations:** 1 Technical University of Munich, TUM School of Medicine and Health, Klinikum rechts der Isar, Research Group MITI, Munich, Germany; 2 TUM School of Medicine and Health, Klinikum rechts der Isar, Department of Surgery, Technical University of Munich, Munich, Germany; 3 TUM School of Computation, Information and Technology, Technical University of Munich, Munich, Germany

**Keywords:** Audio analysis, Minimally invasive surgery, Acoustic emission, Machine learning

## Abstract

**Purpose:**

Acoustic information can contain viable information in medicine and specifically in surgery. While laparoscopy depends mainly on visual information, our goal is to develop the means to capture and process acoustic information during laparoscopic surgery.

**Methods:**

To achieve this, we iteratively developed three prototypes that will overcome the abdominal wall as a sound barrier and can be used with standard trocars. We evaluated them in terms of clinical applicability and sound transmission quality. Furthermore, the applicability of each prototype for sound classification based on machine learning was evaluated.

**Results:**

Our developed prototypes for recording airborne sound from the intraperitoneal cavity represent a promising solution suitable for real-world clinical usage All three prototypes fulfill our set requirements in terms of clinical applicability (i.e., air-tightness, invasiveness, sterility) and show promising results regarding their acoustic characteristics and the associated results on ML-based sound classification.

**Conclusion:**

In summary, our prototypes for capturing acoustic information during laparoscopic surgeries integrate seamlessly with existing procedures and have the potential to augment the surgeon’s perception. This advancement could change how surgeons interact with and understand the surgical field.

## Introduction

The interpretation of acoustic signals has a long history in various medical disciplines. In addition to ultrasound, which creates images using sound waves, audible sounds can also serve as diagnostic tools, e.g., in auscultation for respiratory and cardiovascular diseases, COVID-19 detection [1] and orthopedic disorders via vibroarthographic signals [2]. For decision-making, surgeons rely on various information sources during Minimally invasive surgery (MIS), including preoperative data, procedural knowledge, and, most importantly, intraoperative insights. While visual information is key, also auditory cues from peripheral devices, like HF generators or vital sign monitors, are important. Further enhancement of intraoperative support based on acoustic signals is demonstrated by recent advancements, including work by Seibold et al. deep-learning method with acoustic sensing for drilling breakthrough detection during orthopedic surgery [3]. The team of Illanes et. al. has been researching the use of acoustic emissions(AE) to provide additional feedback during MIS. Their approach, called Surgical Audio Guidance (SURAG), aims to enhance the surgeon’s perception by analyzing the mechanical vibrations generated from the interactions between surgical instruments and tissues. To acquire this data, an audio sensor is attached to the proximal end of the instruments. Proposed applications include, among others, the provision of meaningful haptic information as real-time feedback during robotic-assisted surgery (e.g., tissue surface characteristics) [4] and the extraction of guidance information from AE such as detection of tissue layer crossing, events during needle insertion and manual cutting events [5, 6]. While Illanes et al. focus on vibro acoustic emissions, i.e., analysis of mechanical waves propagating through an instrument during an interaction, our research focuses on the capturing and analysis of airborne sound emissions. These sound emissions predominantly occur also during tissue-tool interactions, e.g., diathermy sounds but are not limited to the tool interaction. Additionally, ambiance sounds like heartbeats, bowel sounds, or hissing sounds of leaking air are audible within the abdominal cavity, although potentially out of sight.

In the following, we are focusing on airborne sound emissions during electrocautery in laparoscopic surgeries. We have previously demonstrated surgeons’ ability to distinguish tissue types based on these emissions and have applied machine learning for automated differentiation [7]. Our aim is to extend these findings to laparoscopic settings, enhancing the potential for acoustic information retrieval and assisting surgeons with new sensory data. In laparoscopic surgeries, the transmission and recording of sounds from inside the abdomen to the external environment is challenging. We aim to transmit these sounds using standard trocars, providing real-time feedback to the surgical team or as input for machine learning analysis. We developed and evaluated several prototypes for this purpose, focusing on clinical applicability, medical safety, and sound transmission quality. The most promising prototypes were further evaluated for their feasibility in machine learning-based processing to optimize the utilization of acoustic information in laparoscopic surgeries.

## Methods

### Development of prototypes

During the conceptual phase, we defined several requirements for the design of trocar adapters derived from a requirement analysis with leading clinical experts in the field of MIS, resulting in the four prioritized requirements:

1. **Sterility**: The adapter must be applicable in a sterile surgical environment.
2. **Invasiveness**: The adapter should not increase access trauma.
3. **Airtightness**: The adapter must remain airtight to maintain a pneumoperitoneum at usually applied pressures up to 15 mmHg.
4. **Acoustic Transmission**: The adapter should exhibit minimal attenuation or damping of the acoustic signal and robustness against structure-born and airborne acoustic noise, allowing for machine learning (ML)-based classification, i.e., the alteration of sound may not prevent the classification of diathermy sounds using spectrograms.

As a signal transducer, we chose the DPA CORE 4660 Heavy Duty microphone for sound recording due to its omnidirectional pattern, durability, and compact size (5.4 mm capsular head diameter). It is well suited for integrating into space-constrained designs and served as a starting point for the different concepts. We utilized 3D-printing to rapidly iterate through various concepts and assess them for clinical applicability, airtightness, and acoustic properties. Hereby, we narrowed the potential solutions down to three main prototypes.

#### Dual-channel insert

The concept for the dual-channel insert is designed similarly to a reduction sleeve and fits into a 13.5 mm trocar, effectively splitting it into two separated 5 mm channels. One channel is designated for laparoscopic instruments, while the other is allocated for the sterile-draped microphone (see 2.2.1 *Sterility*). Inserted parallel to the instrument, it extends to the distal opening of its channel and records direct sound from the intraperitoneal cavity.

The microphone is covered sterile and inserted parallel to the instrument, extending to the distal opening of the channel to capture sounds directly from the intraperitoneal cavity. The airtightness for the two channels is ensured by the following design: The 5 mm instrument channel is sealed by accommodating a standard multifunctional valve from Karl Storz SE & Co. KG for standard 6 mm trocars via bayonet mount. For the microphone channel, a dedicated flange with a circular silicone sealing (SF00: Shore Hardness 00 ShA) was designed, enabling an airtight passage for the microphone.

#### Luer-Lock adapter

This adapter is designed to be connected to the Luer-Lock connector of a trocar. Typically, this port is employed for CO_2_ insufflation. Given the flow of gas between the connector on the trocar to the insufflated cavity, this passage can also serve to capture airborne sound waves. This adapter features a Luer-Lock thread leading to a cavity designed for microphone insertion. To reduce the transmission of structural-borne noise, the microphone is decoupled from the adapter housing by embedding the microphone in a silicone sleeve. An additional silicon lid seals the entry point for the microphone’s cable. The inner and outer seals were crafted from silicone (00 ShA) using 3D-printed molds. To ensure sterility, the microphone is draped sterile (see 2.2.1). To further minimize noise, it is optimal to connect this adapter to a trocar where instrument changes are rare.

#### Silicone tube

This concept also utilizes the Luer-Lock port: a 2 m silicone tube is connected to a trocar’s Luer-Lock port with the microphone positioned at the tube’s distal end. This arrangement keeps the microphone outside the sterile field while capturing sound waves traveling through the tube. To prevent the potential flow of particles or liquids to the trocar, the microphone end is positioned lower than the trocar, eliminating the need for a sterile microphone cover. The interface between the microphone and the tube is airtight due to a press fit. This setup should be connected to a trocar with minimal instrument changes to minimize noise interference.

#### Reference microphone

For the experimental evaluation of the different prototypes described above a benchmark or reference measurement is essential. For this purpose, a microphone enclosed in a sterile ultrasound cover is positioned inside a standard trocar with the capsule protruding beyond the distal tip of the trocar. To ensure airtightness and prevent noise interference from escaping gas, the trocar’s outer port is sealed. A specialized flange has been designed that allows the snug passage of the microphone cable. It is to note, that this design serves solely for evaluation purposes and does not propose a potential solution to our primary problem statement—mainly due to invasiveness, since it would require an additional access incision.

### Evaluation of prototypes

**Fig. 1:**
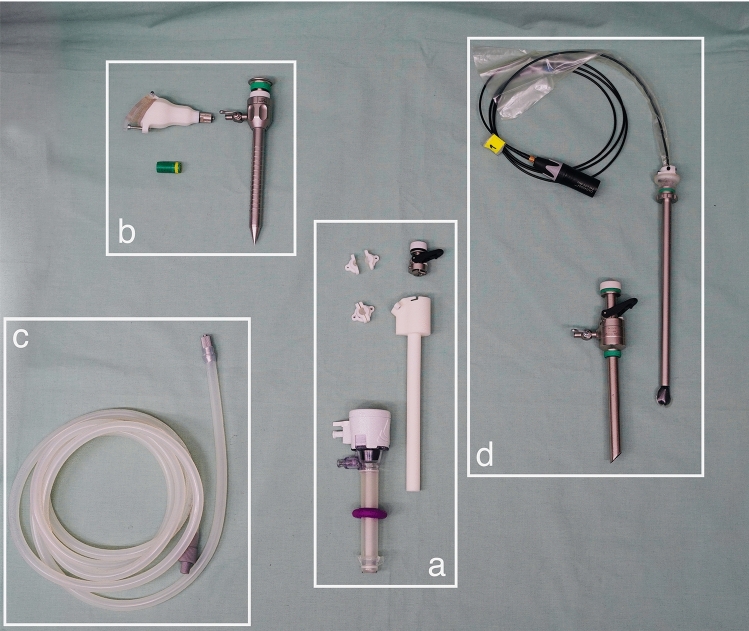
Overview of different prototypes for evaluation: **a** dual-channel insert, **b** Luer-Lock adapter, **c** silicon tube, **d** reference microphone covered by ultrasound probe sleeve

In the following section, we describe the evaluation steps for each prototype depicted in Fig. 1. We will systematically assess the performance against four key requirements as outlined in 2.1: sterility, invasiveness, airtightness, and acoustic transmission. The assessment of the applicability of spectrogram-based classification will be explained in Sect. 2.3.

#### Evaluation of sterility and invasiveness

Defining clear and objective metrics to assess the level of sterility and invasiveness in the context of our prototypes is not trivial. Although they are distinct in their nature, we have chosen to use a qualitative assessment for both requirements. In the following, we will explain the concepts of ensuring sterility and minimizing invasiveness.

*Sterility* The assurance of sterility in a surgical setting is of utmost importance, which we try to ensure through three approaches:

1. Microphone covering: Since the chosen microphone would not withstand steam sterilization, it is only used draped in a sterile sleeve within the sterile field. This prevents any direct contact between the sterile surgical field and non-sterile surfaces of the microphone. We conducted a comparison of three different options for the sleeve: a) a sterile glove, b) a protective sterile cover for extracorporeal ultrasound transducers, and c) a sterile sleeve for intracorporal laparoscopic ultrasound transducers. Similar to the approach described in 2.2.3, we characterized the three covers based on their recorded power spectral density when exposed to pink noise. Showing the least dampening across the frequency range, we selected the laparoscopic ultrasound cover from ECOLAB made of polyurethane (model Ultracover $10\times 400$ mm; Art. No.: 16565).
2. Biocompatible Material: All adapters, except the silicone tube, are manufactured via 3D printing. For their application during sterile surgery, the adapters can be printed with biocompatible and autoclavable material. We verified this approach using the Fromlabs Form 3B printer with *BioMed Clear Resin*, which is certified by ISO 18562.
3. Remote Placement: By positioning the microphone at a distance from the incision (i.e., in case of the Luer-Lock adapter) or even further away from the surgical field (i.e., in the case of the silicone tube). This separation helps eliminate direct microbial traffic from the microphone to the surgical site and inherently promotes sterility.

While quantifying sterility through objective metrics like microbial counts might be ideal, it falls outside the scope of our current evaluation. Nevertheless, the aforementioned approaches align with conventional methods applied in surgical environments to maintain sterility.

*Invasiveness* While quantifying invasiveness would ideally involve surgical outcome metrics, our current evaluation focuses only on design factors. All prototypes utilize standard trocars, routinely used in minimally invasive surgeries, limiting our invasiveness assessment to the impact on trocar sizes. This aspect is only relevant for the Dual-Channel Insert, which requires a 13.5 mm trocar which is larger than the typical 5 mm instrument trocars. However, this trocar size is still in the range of standard trocars and is also used for larger (3D-)laparoscopes and might be still acceptable for clinical routine. The other two designs utilize the Luer-Lock connector and achieve universal compatibility: They are not constrained by trocar dimensions and can be attached to any trocar used during surgery without affecting invasiveness.

#### Evaluation of airtightness

Typical CO_2_ leakage rates of 200 to 400 mL/min during laparoscopy to maintain the pneumoperitoneum at [ to range-units = single]1015mmHg are reported in the literature [8]. We targeted a leakage rate of at least one order of magnitude lower than this while increasing the pressure and defined acceptance criteria of 20 mL/min, i.e., 10 mL per 30 s at 50 mmHg. Testing of our prototypes was performed as specified in ISO 80369-7 using a pressure gauge GMH 3156 (GSM Messtechnik GmbH) equipped with an *MSD 1 BRE* sensor. A 3-way valve connected a syringe, the pressure sensor, and the unit under testing. A dedicated adapter was designed for the flange of the dual-channel insert, compensating for a missing Luer-Lock connection. Each test was initiated with 50 mL of air in the syringe and increased the pressure until it reached 50 mmHg. The pressure was maintained for 30 s. We used a 3-way valve to connect a syringe, the pressure sensor, and the unit under testing. A dedicated adapter was designed for the flange of the dual-channel insert, compensating for a missing Luer-Lock connection. We initiated each test with 50 mL of air in the syringe and increased the pressure until it reached 50 mmHg and was maintained for 30 s. The syringe was then released until the pressure gauge read 0 mmHg. The difference to 50 mL was recorded as leakage. We repeated this test five times for each prototype.

#### Evaluation of acoustic characteristics

One key objective of our study is to determine the impact of the different prototypes on sound transmission, which is done via the evaluation of the individual transfer functions (TFs). For the transfer characteristics of a linear time-invariant (LTI) system, it is of interest to determine the amplification or attenuation of an input signal based on its frequency. The TF describes these linear transfer characteristics of any LTI system in a given frequency range. It is calculated using the Fourier transform of the impulse response. For any given input signal *s*(*t*) that passes through the LTI, the output *g*(*t*) can be calculated as the convolution with the impulse response *h*(*t*) as . Hence the system’s TF $H(\omega )$ results from the complex quotient as: $H(\omega ) = \frac{G(\omega )}{S(\omega )}$.

In our experiments, the impulse response was obtained using MATLAB’s *Impulse Response Measurer* (R2020b, Audio Toolbox). We used a professional audio interface (Steinberg UR824) which allows simultaneous generation and recording of audio signals via a USB-connected Laptop controlled by MATLAB. A combination of a small HIFI speaker and amplifier was connected to the audio interface’s output. In an attempt to simulate a laparoscopic setting, the speaker was placed within a surgical laparoscopy trainer (*ELITE* trainer, CLA, Coburger Lehrmittelanstalt) while the reference microphone (DPA Core 4660) was inserted through a trocar port and was connected as an audio input to the interface. We ensured a low noise level ($-30$ dB) when no output signal was played by using the *Excitation Level Tool* and white noise to level in-/output gains during setup.

To acquire the impulse response (IR), the maximum length sequence (MLS) method was chosen, which uses a pseudo-random signal resembling the stochastic properties of white noise. The resulting IR is derived by a circular cross-correlation [9]. The MLS covers frequencies from 10 Hz to 22 kHz. With a sampling frequency of 48 kHz, the Nyquist-Shannon theorem is met. The acquisition of the IR was repeated two times for each prototype with two different trocars (11 mm and 13.5 mm except the dual channel, which only applies to 13.5 mm, with and without an instrument inserted (see also first column, i.e., test set categories of Table 2). This results in a set of 18 measurements for the prototypes supplemented by two reference measurements.

To compare the resulting TFs of the prototypes, we calculate the relative difference to the reference microphone’s impulse response:

1 $$\begin{aligned} \varDelta H(\omega ) = 20 \cdot \log _{10} \left( \frac{\left| H_{Prototype}(\omega )\right| }{\left| H_{ref\_Mic}(\omega )\right| } \right) \end{aligned}$$

where $\varDelta H(\omega )$ is the *relative transfer function*, $H_{Prototype}$ and $H_{ref\_Mic}$ the TF of the individual prototype and the reference microphone, respectively. The resulting difference in dB of the TF $\varDelta H(\omega )$ is used to quantify the magnitude differences in the frequency response of the prototypes relative to the reference microphone.

Since some noise was observed for frequencies above 5 kHz on the resulting relative TFs, we applied a Savitzky-Golay filter with a relative bandwidth of 1/6 octave for better comparability.

### Applicability of spectrogram-based classification

In a previous study [7], we developed a deep-learning model for the classification of tissue-specific sounds during diathermy. The setup required placing a draped microphone directly into the abdominal cavity which is not suitable for clinical use due to concerns about invasiveness. To address this limitation, the primary focus of this study is to enable the capturing of abdominal sound in real-world laparoscopic settings by using the newly developed adapters. The secondary focus is to demonstrate the feasibility of the machine-learning-based sound classification with these new adapters and their acoustic characteristics. Therefore we aim to adapt our classification model to the acoustic characteristics of each prototype. This adaptation of the model to the adapters is carried out in three main steps, as described in more detail in the subsequent sections: first, replacing the original model with a different, state-of-the-art model for sound classification. Second, the convolution of the dataset with the adapter-specific IR and third, the fine-tuning of the model to the acoustic characteristics.

#### Audio classification model

Instead of utilizing the previous model from [7] one to one, we decided to adopt a different network architecture, called YAMNet (“Yet another Audio Mobilenet Network”), an audio event classifier, which is based on the MobileNet v1 architecture. It is pre-trained on the AudioSet Corpus [10] comprising of more than 5000 h of audio segments across 527 audio event categories. By utilizing transfer learning for a network that was pre-trained on a large audio dataset, we presume to achieve better robustness against noise as demonstrated in [11].

Consequently, we fine-tuned the pre-trained YAMNet on our original dataset acquired in [7], i.e., on the cutting and coagulation sounds during diathermy on different tissue types, i.e., liver, muscle, fat, fascia, and an idle class for the absence of any diathermy sounds. The dataset consists of 1620 samples, each varying in length from 0.76 s to 5.17 s for tissue types and a maximum length of 140 s for samples of the *idle*-class. Since the audio samples of the *idle*-class are usually longer (as they correspond to the pauses between Diathermy events), the dataset was balanced by limiting the accumulated audio duration for the *idle*-class to the maximum accumulated duration of one of the other classes. We then split the dataset at a ratio of 80:10:10 for training-, validation-, and test sets, respectively. Each audio signal is pre-processed by the following steps:

1. Homogenization in terms of channels and sampling frequency, i.e., conversion to monophonic and re-sample to 16 kHz
2. Application of a high-pass filter at 100 Hz
3. Zero-padding for audio signals shorter than 0.98 s
4. Segmenting the audio signal in consecutive windows of 0.98 s length with a 75% overlap.
5. Transformation of each segment into a log-scaled mel spectrogram. This was done using a short-time Fourier transform (STFT) with overlapping Hanning windows of 25 ms with a 10 ms hop-length and a subsequent application of the 64-band mel frequency filter banks.

**Fig. 2:**
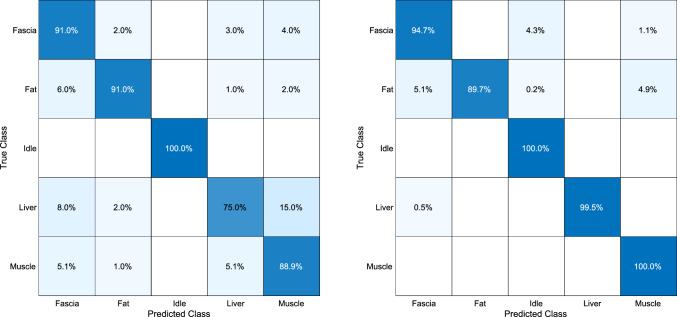
Comparison of classification accuracy. Left: Confusion matrix for results in [7] vs. right: results for newly implemented classification

The resulting mel spectrograms are stored as matrices of $96\times 64 \times K$, with each mel spectrogram consisting of 96 frames of each 25 ms representations. The 64 represents the mel bands from 125 Hz to 7500 Hz, and *K* represents the number of spectrogram images determined by the audio input length and the overlap (75%), resulting in the following number of spectrograms (i.e., $\sum {K}$): 10234 for training, 1490 for validation, and 1517 for testing. We reduced the output layer of YAMNet from the original 521 classes to 5 classes and re-trained the network. To determine an optimal parameter set for the transfer learning, we performed a grid-search across multiple hyper-parameters: optimization algorithm {ADAM, RMSprop}, learning rate: 1e-3, 1e-4, 1e-5, mini-batch size 64, 128, 256. For each parameter set, the resulting model was selected based on the best validation loss during training and then applied to the test set. The best test performance was achieved by the following parameter set: RMSProp optimizer with a constant learning rate of $1\times 10^{-4}$ and a mini-batch size of 128. Hereby, the network achieves a mean accuracy (mAcc) of 0.966, with a mean average precision (mAP) of 0.943 and mean average recall (mAR) of 0.968, leading to a F1-score of 0.955, which surpasses results from our previous results in [7] as shown in the in Fig. 2.

#### Convolution of the audio set

To avoid generating a new dataset for each adapter, we convolved the original audio samples with the measured IR of each adapter, simulating the acoustic response as if the sounds were recorded directly with the adapter. This approach allows us to efficiently assess the classification accuracy for each prototype without the need for extensive data collection.

The convolution operation can be mathematically expressed as:

2 $$\begin{aligned} y[n] = (f * g)[n] = \sum _{j=max(0, k-N+1)}^{min(k, M-1)} f[j]g[n-j] \end{aligned}$$

where *y* is the output signal, *f* is the audio signal with length *M*, and *g* is the impulse response of the adapter, with a length of *N*.

Subsequently, we assessed and compared the classification accuracy of both the directly recorded sounds and their convoluted counterparts. Our initial findings showed that the YAMNet model, trained on the original dataset, yielded only a modest performance with an average accuracy of $0.6207 \pm 0.1331$ across all adapter-specific convoluted test sets. As a consequence, we fine-tuned the model for each adapter on a respective convoluted training set to improve its performance. For the fine-tuning we applied the same hyper-parameter set as described in 2.3.1. The performance results for the fine-tuned models are listed in 2.

## Results

We evaluated the leak-tightness for the final versions of the three prototypes under the pressure of 50 mmHg for a period of 30 s. The dual-channel insert prototype showed a loss of 2.4 mL ± 0.49 mL, which is considered low enough to meet the requirements. For the Luer-Lock adapter prototype a loss of 1.6 mL ± 0.49 mL was measured. The silicone tube prototype demonstrated the highest leak-tightness with a loss of 0.6 mL ± 0.49 mL.

As described in 2.2.3, we determined the impulse response for each prototype using the *Maximum Length Sequence* (MLS) technique and then derived the respective transfer function. We calculated the relative TFs for the three prototypes by subtracting each prototype’s TFs from the reference microphone’s TF, as outlined in equation (1). This comparison uses the TFs with an instrument inserted through the trocar to simulate surgical conditions.

**Fig. 3:**
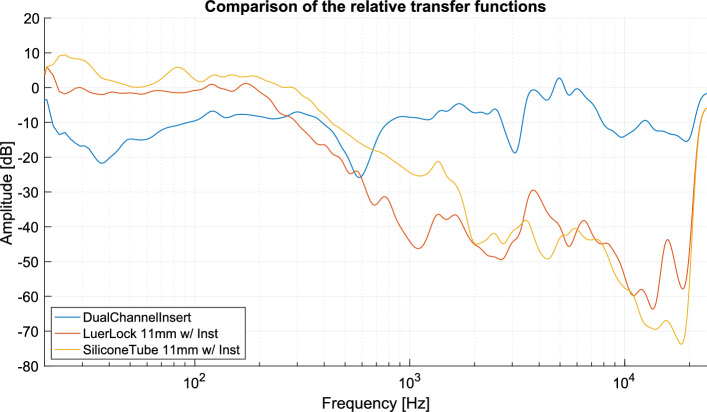
Relative transfer functions of the three prototypes *Dual-channel insert* (blue), *Luer-Lock adapter *(red) and *silicone tube* (yellow), with the latter two attached to a 11 mm trocar with an instrument inserted

Figure 3 shows the respective filtered relative TFs $\varDelta H(\omega )$. The transfer characteristics and, hence, the relative transfer function of the two prototypes, Luer-Lock adapter and silicone tube, exhibit similar patterns. A steady decline in frequency response occurs at 200 Hz for the Luer-Lock adapter and 300 Hz for the silicone tube prototype. The frequency response of the silicone tube prototype is approximately 10–20 dB higher than that of the Luer-Lock adapter prototype in the region around 300 Hz to 1000 Hz. The attenuation increases significantly to about $-40$ dB compared to the reference microphone at about 1 kHz. The response of the dual-channel insert, due to its design, is closest to that of the reference microphone with an average attenuation of 9.4 dB. A comparative summary of the results is presented in Table 1.

**Table 1:** Comparison of key characteristics of the three prototypes

Criteria	Dual-channel insert	Luer-lock adapter	Silicone tube
Air-tightness (leakage @ 50 mmHg for 30 s)	2.4 mL ± 0.49 mL	1.6 mL ± 0.49 mL	0.6 mL ± 0.49 mL
Invasiveness	Requires 13.5 mm trocar (more invasive)	Fits any trocar size (minimally invasive)	Fits any trocar size (minimally invasive)
Sterility	Sterile-draped mic in dedicated channel	Sterile-draped mic, autoclavable adapter	Mic outside sterile field, no covering needed
Acoustic characteristics	Closest to reference mic, average attenuation 9.4 dB	10–20 dB lower than reference microphone from 300–1000 Hz	Weakest performance, 40 dB attenuation @ 1 kHz

*Results on spectrogram-based classification* As described in 2.3, we first tested the convoluted dataset for each prototype on the YAMNet model trained on the dataset obtained via the reference microphone. The results for the test data are shown in the second column of Table 2 referred to as the *original model*. In a second step, we fine-tuned the YAMNet model for each prototype on its respected convoluted dataset to account for the change in acoustic response. The resulting classification metrics for the fine-tuned models are shown in columns three to six.

**Table 2:** Overview of classification results for the original test set (first row) and its variances convoluted by IR for the original model (second column) and the fine-tuned version (third column)

Test set (IR conv.)	mAcc for orig. model	mAcc f-t. model	mAP f-t. model	mAR f-t. model	F1-score f-t. model
original (ref.Mic)	0.9657	n/a	n/a	n/a	n/a
Dual channel	0.9097	0.8952	0.8804	0.9271	0.9032
Luer-Lock 11 mm w/o I	0.5939	0.9598	0.9590	0.9494	0.9542
Luer-Lock 11 mm w/ I	0.4061	0.9314	0.9290	0.9348	0.9319
Luer-Lock 13 mm w/o I	0.6941	0.9420	0.9224	0.9207	0.9216
Luer-Lock 13 mm w/ I	0.5900	0.9123	0.8789	0.9321	0.9047
Tube 11 mm w/o Instr	0.6236	0.8134	0.8299	0.8496	0.8397
Tube 11 mm w/ Instr	0.5682	0.8504	0.9020	0.8936	0.8978
Tube 13 mm w/o Instr	0.5728	0.9268	0.9118	0.9261	0.9148
Tube 13 mm w/ Instr	0.6282	0.7416	0.7888	0.7594	0.7738

Values for mean accuracy (mAcc), mean average precision (mAP), mean average recall (mAR), and F1-scores are shown only for the fine-tuned models

## Discussion

While the testing in real surgical environments is pending due to the lengthy ethical approval process, the prototypes have shown high potential in controlled settings. The *dual-channel insert* prototype can be inserted into any regular 13.5 mm trocar, functioning similarly to a trocar reduction sleeve. Additionally to the instrument, the insert provides access to the intraperitoneal cavity through a second insertion channel for a sterile covered microphone to directly record acoustic signals. Due to its design, it has a transfer function matching closely to our reference microphone, suggesting a high potential for precise, unaltered sound transmission. This similarity in sound transmission characteristics is also reflected in the classification results for the *original model* on the dual-channel convoluted test set, with a $\text {mAcc}>90\%$ (see Table 2 ).

The other two designs, i.e., *Luer-Lock adapter and silicone tube* connect to the trocar’s Luer port, which is typically used for insufflation and hence presents an air passage to the intraperitoneal cavity through the trocar. Sound travels from the peritoneal cavity through the prototypes to the microphone without being inserted into the cavity. This connection is available on any trocar, independent of the manufacturer, allowing for the potential widespread use of these concepts.

The *Luer-Lock Adapter* is designed to directly mount a microphone onto the Luer port. Friebe et al. proposed the connection of their sensing unit on laparoscopic tools directly or on trocars indirectly via Luer-Lock connectors [12]. While their sensing unit aims for the acquisition of vibro-acoustic signals, the Luer-Lock adapter proposed here is specifically designed for the acquisition of airborne sounds. Being in the sterile surgical field requires the microphone to be encased in a sterile sleeve and the autoclavable adapter to be assembled in-field. While this concept affects the surgical workflow by a few additional steps, its clinical acceptability is yet to be further evaluated, especially since the adapter will increase the weight by 39.5 g. Notably, the Luer-Lock adapter has achieved high classification accuracy with mAcc scores of over 90%.

The *Silicone Tube* also connects to the Luer port but is designed to capture the sound traveling from the Luer port through a sterile tube from the surgical field to the microphone connected to the tube outside of the surgical field.

The silicone tube adapter has displayed a strong attenuation, resulting in the weakest performance in terms of classification accuracy when compared to the other two adapters. However, the results are still acceptable, with an mAcc between 74–93% for the fine-tuned models. The adapter is notable for its robust sterility concept and minimal invasiveness, as it doesn’t cause any additional trauma, which makes it highly compatible with surgical needs. Leveraging an insufflation tube, a commonly used asset in laparoscopic surgery, the adapter requires minimal adjustments to the standard surgical workflow, enabling a potential seamless integration. Its lightweight design ensures no additional strain on the abdominal wall, further minimizing trauma.

Generally, each of the three prototypes demonstrated promising results regarding airtightness, with air leak volumes ranging between 0.6 mL and 2.4 mL over a 30 s period. Given the typical insufflation flow rates of 200 to 400 mL/min, this level of leak tightness satisfactorily meets our set requirements of less than 20 mL/min. Considering the classification accuracy, we demonstrated that the new classification model yielded robust performance after fine-tuning the model to the respective acoustic characteristics of each prototype.

Finally, we would like to acknowledge the limitations of the study. The lack of real surgery testing means that while our prototypes function promisingly in controlled environments, their performance under actual surgical conditions remains to be determined. Further studies, following ethical approval, are required to conclusively validate the reliability and usefulness of these adapters in surgery.

## Conclusion

Our study introduces a novel approach for the monitoring of intraperitoneal airborne sound during laparoscopic surgery. Each of the three prototypes–dual-channel insert, silicone tube, and Luer-Lock adapter caters to different surgical needs, balancing sound fidelity, invasiveness, and workflow integration

In direct comparison, the *dual-channel insert* shows superior acoustic performance but requires a larger trocar, increasing invasiveness. The *silicone tube*, while non-invasive and offering a robust sterility concept, compromises on sound quality due to higher dampening. The Luer-Lock adapter, positioned directly at the trocar, offers a middle ground in terms of sound dampening but complicates the surgical workflow with its installation process and added trocar weight.

The choice of a particular concept would rely on the specific application in the clinical setting, i.e., whether the focus is on machine learning-based sound analysis or unaltered abdominal sound monitoring. Further applications could involve the exploration of sonification concepts [13], where the captured acoustic information can be transformed into more perceptual meaningful forms. By mapping acoustic signals to auditory cues that are easier to interpret, surgeons could gain enhanced situational awareness, enhancing their visual observations. We believe that this work can enhance surgeons’ perception and change how they interact with the surgical field. Continued refinement of the ML-based sound classification and clinical evaluation could lead to enhanced assistance systems supporting tissue differentiation, thereby making surgeries safer and more efficient. In the context of robotic surgery, these advancements could support the development of safe partial-autonomous and autonomous surgical tasks by adding another low-level information modality.

## Data Availability

The data used in this study can be provided upon reasonable request.
